# A blood-based diagnostic test incorporating plasma Aβ42/40 ratio, ApoE proteotype, and age accurately identifies brain amyloid status: findings from a multi cohort validity analysis

**DOI:** 10.1186/s13024-021-00451-6

**Published:** 2021-05-01

**Authors:** Tim West, Kristopher M. Kirmess, Matthew R. Meyer, Mary S. Holubasch, Stephanie S. Knapik, Yan Hu, John H. Contois, Erin N. Jackson, Scott E. Harpstrite, Randall J. Bateman, David M. Holtzman, Philip B. Verghese, Ilana Fogelman, Joel B. Braunstein, Kevin E. Yarasheski

**Affiliations:** 1 C2N Diagnostics, 20 S Sarah Street, St. Louis, MO 63108 USA; 2 Department of Neurology, Hope Center for Neurological Disorders, Knight Alzheimer’s Disease Research Center, Washington University School of Medicine, St. Louis, MO 63110 USA

**Keywords:** Alzheimer’s disease, Plasma biomarkers, Neurodegeneration, mass spectrometry

## Abstract

**Background:**

The development of blood-based biomarker tests that are accurate and robust for Alzheimer’s disease (AD) pathology have the potential to aid clinical diagnosis and facilitate enrollment in AD drug trials. We developed a high-resolution mass spectrometry (MS)-based test that quantifies plasma Aβ42 and Aβ40 concentrations and identifies the ApoE proteotype. We evaluated robustness, clinical performance, and commercial viability of this MS biomarker assay for distinguishing brain amyloid status.

**Methods:**

We used the novel MS assay to analyze 414 plasma samples that were collected, processed, and stored using site-specific protocols, from six independent US cohorts. We used receiver operating characteristic curve (ROC) analyses to assess assay performance and accuracy for predicting amyloid status (positive, negative, and standard uptake value ratio; SUVR). After plasma analysis, sites shared brain amyloid status, defined using diverse, site-specific methods and cutoff values; amyloid PET imaging using various tracers or CSF Aβ42/40 ratio.

**Results:**

Plasma Aβ42/40 ratio was significantly (*p* < 0.001) lower in the amyloid positive vs. negative participants in each cohort. The area under the ROC curve (AUC-ROC) was 0.81 (95% CI = 0.77–0.85) and the percent agreement between plasma Aβ42/40 and amyloid positivity was 75% at the optimal (Youden index) cutoff value. The AUC-ROC (0.86; 95% CI = 0.82–0.90) and accuracy (81%) for the plasma Aβ42/40 ratio improved after controlling for cohort heterogeneity. The AUC-ROC (0.90; 95% CI = 0.87–0.93) and accuracy (86%) improved further when Aβ42/40, ApoE4 copy number and participant age were included in the model.

**Conclusions:**

This mass spectrometry-based plasma biomarker test: has strong diagnostic performance; can accurately distinguish brain amyloid positive from amyloid negative individuals; may aid in the diagnostic evaluation process for Alzheimer’s disease; and may enhance the efficiency of enrolling participants into Alzheimer’s disease drug trials.

**Supplementary Information:**

The online version contains supplementary material available at 10.1186/s13024-021-00451-6.

## Background

Alzheimer’s disease (AD) is the most common form of dementia [1]. Globally, nearly 50 million people have AD or a related dementia, yet only 25% of people living with AD have been diagnosed [2, 3]. Currently, AD affects 5.8 million Americans 65 years and older, and by 2050, AD prevalence in the US is expected to increase to 13.8 million [1]. AD is a progressive, irreversible degenerative condition that affects a person’s memory, cognitive abilities, and personality. AD dementia is associated with increased disease susceptibility in organs outside the brain in ways that can ultimately lead to death. In the US, AD is the sixth-leading cause of death.

With several promising AD-modifying therapies in development, early detection of brain amyloidosis will be imperative for selecting and treating patients. Current AD diagnostic guidelines include tests that detect the presence of brain amyloid-β (Aβ) plaques using either amyloid PET imaging or low cerebrospinal fluid (CSF) Aβ42 levels or Aβ42/40 ratio; biomarkers for dysregulated Aβ metabolism and plaque formation [4–10]. Although amyloid PET imaging and CSF biomarkers have significantly improved the detection of brain amyloidosis, there is still a critical need for safe, lower cost, less resource-intensive, broadly available, blood-based biomarkers that identify the presence or absence of brain amyloid plaques. Herein, we describe the first generation of a Mass Spectrometry (MS)-based blood test that addresses this critical need.

Substantial effort and resources have been devoted to quantifying blood biomarkers (e.g., Aβ42, Aβ40) as potential proxies for brain amyloid plaques [11–24]. Using traditional enzyme-linked immunoassay (ELISA) technology, most prior studies found poor concordance between blood Aβ concentrations and either brain amyloid status or AD [25]. Recently, mass spectrometry-based technologies have gained traction as highly sensitive and specific methods for quantifying Aβ isoforms in CSF and blood samples [17, 20, 21, 26–31]. These studies found that low plasma Aβ42/40 concentration ratios (or high plasma Aβ40/42 concentration ratios [21]) are associated with the presence of brain amyloid plaques. Interestingly, a low plasma Aβ42/40 ratio identified the presence of brain amyloid plaques prior to the onset of a positive amyloid PET scan [17]. During the research and development process, we designed a streamlined, high throughput, liquid chromatography-tandem mass spectrometry (LC-MS/MS) analytical platform that quantifies plasma Aβ40 and Aβ42 levels and identifies plasma Apolipoprotein E (ApoE) isoform-specific peptides.

To establish proof-of-principle, we tested the robustness, clinical accuracy, and commercial viability of this novel LC-MS/MS assay by evaluating concordance between LC-MS/MS-based measures of plasma Aβ42/40 concentration ratio, ApoE phenotype, and the presence or absence of brain amyloidosis determined using CSF and amyloid PET imaging biomarkers among participants enrolled by multiple memory clinics and academic research centers across the US.

## Methods

### Participants and samples

We obtained random, banked plasma samples collected from six independent cohorts that were collected from participants enrolled in site-specific clinical studies, where brain amyloid status (positive, negative, SUVR) was determined using either amyloid PET imaging or CSF biomarker analysis (Table 1). Plasma samples were collected, and brain amyloid status was determined using site-specific, but diverse methods and protocols. In this retrospective sample analysis, control of pre-analytical conditions during blood sample collection and processing was not possible. For example, five of the six sites provided plasma from blood collected in K_2_ EDTA tubes, and one site (cohort 2) used lithium heparin tubes. Other than the collection tube, blood sample processing procedures were similar among the different sites. Briefly, blood samples were centrifuged at room temperature or 4 °C within 30–60 min of phlebotomy. Plasma was aliquoted (0.5–1.0 mL) into polypropylene tubes and frozen at − 70 to − 80 °C within 2 h of phlebotomy. All samples were deidentified, shipped on dry ice to C_2_N, and analyzed in a blinded manner. Some sites obtained brain amyloid PET status using Pittsburgh compound B (PiB) while other sites used FDA-approved amyloid tracers (Amyvid, NeuraCeq); and some sites used CSF Aβ42/40 concentration ratios, different quantification methods (ELISA, MS), and different cutoff values to assign amyloid positivity or negativity. Most but not all sites provided the same demographic and phenotype data for their cohorts (Table 1). Sites are referred to as cohort 1–6. A total of 414 participants’ plasma samples were received, prepared and analyzed using the following sample preparation and liquid chromatography-mass spectrometry methods.

**Table 1:** Cohort demographics and plasma biomarker values

	Cohort 1 (***N*** = 37)	Cohort 2 (***N*** = 94)	Cohort 3 (***N*** = 121)	Cohort 4 (***N*** = 26)	Cohort 5 (***N*** = 96)	Cohort 6 (***N*** = 40)	All Cohorts (***N*** = 414)
**Age**							
Mean (SD)	73.1 (7.8)	71.1 (8.0)	66.1 (7.8)	75.1 (6.8)	71.4 (7.6)	69.7 (9.1)	70.0 (8.3)
Min	59.2	56.7	47.0	66.1	45.0	46.1	45.0
Max	87.5	93.1	85.0	88.9	89.0	86.0	93.1
**Sex**							
Male (%)	54.1%	58.5%	23.1%	57.7%	40.6%	35%	41.3%
**Amyloid Positive (%)**	49%	46%	17%	42%	49%	52%	39%
**CDR**							
Percent CDR = 0 [N]	- [0]	50% [94]	- [0]	56% [25]	- [0]	75% [40]	57.2% [159]
**MMSE**							
Percent MMSE = 27–30 [N]	45.9% [37]	- [0]	100% [121]	64% [25]	- [0]	85% [40]	84.3% [223]
**Biomarker Used to Determine Brain Amyloid Status (% participants)**							
PiB	–	–	68%	54%	–	52%	28%
Amyvid	–	–	32%	–	22%	48%	19%
Neuraceq	–	–	–	–	78%	–	18%
CSF ELISA	–	100%	–	–	–	–	23%
CSF IPMS	100%	–	–	46%	–	–	12%
**ApoE Proteotype**							
E2/E3 (%)	5.4%	8.5%	5.0%	23.1%	7.3%	7.5%	7.7%
E2/E4 (%)	2.7%	6.4%	–	–	2.1%	5.0%	2.7%
E3/E3 (%)	51.4%	46.8%	47.9%	46.2%	52.1%	45.0%	48.6%
E3/E4 (%)	29.7%	28.7%	29.8%	19.2%	34.4%	35.0%	30.4%
E4/E4 (%)	10.8%	9.6%	17.4%	11.5%	4.2%	7.5%	10.6%
**C**_**2**_**N Plasma Aβ42/40**							
Mean (SD)	0.089 (0.012)	0.102 (0.010)	0.101 (0.009)	0.096 (0.009)	0.087 (0.009)	0.100 (0.009)	0.097 (0.011)
Min	0.065	0.084	0.080	0.082	0.059	0.080	0.059
Max	0.113	0.148	0.126	0.114	0.113	0.117	0.148

No patients had the E2/E2 genotype. Cohort 2 used Heparin tubes whereas all other cohorts used EDTA tubes for blood collection. [N] is the number of participants where data was available/provided

*CSF* cerebrospinal fluid, *ELISA* enzyme-linked immunoassay, *IPMS* immunoprecipitation-mass spectrometry, *CDR* Clinical Dementia Rating, *MMSE* Mini-Mental State examination

### Plasma Aβ calibrators and internal standard preparation

Amino acid analysis (AAA) was performed on full length ^14^N- and uniformly labeled ^15^N-Aβ40 and ^15^N-Aβ42 proteins (rPeptide, Watkinsville, GA) to confirm their chemical purity and the amount of each to be used when preparing calibrator stock solutions. Calibrators were prepared by spiking known incremental amounts of recombinant ^14^N-Aβ40 and ^14^N-Aβ42 into 2% (w/v) recombinant human serum albumin (HSA) in phosphate buffered saline (PBS) that contained a known amount of ^15^N-Aβ40 and ^15^N-Aβ42 proteins. Seven calibrator concentrations were prepared based on the expected physiological range for plasma Aβ proteins: Aβ40 = 24.3–1558 pg/mL; Aβ42 = 3.6–235 pg/mL. A matrix blank prepared from 2% HSA was also included in every analysis.

All participant plasma samples, calibrators, and quality control samples were treated identically throughout sample processing and analysis. To each 450 μL plasma sample, 9 μL of 2.5% (w/v) Tween-20, 23 μL of PBS, 45 μL of 5 M guanidine, and 10 μL of protease inhibitors were added. Final concentrations of full-length ^15^N internal standard proteins were 200 pg/mL and 30 pg/mL for Aβ40 and Aβ42, respectively. After the addition of immunoprecipitation buffers, 40 μL of a slurry containing monoclonal anti-Aβ13–28 antibody (HJ5.1) conjugated to magnetic beads (Dynabeads M-270 Epoxy, Thermo-Fisher) was added to each sample and incubated at room temperature for 90 min. After incubation, the magnetic beads were washed with PBS and triethylammonium bicarbonate (TEABC) to remove non-specific binding contaminants prior to sample digestion. Bound amyloid proteins were digested at 37 °C in 100 μL of TEABC containing 0.94 ng/μL lysN metalloprotease (MS Grade, Pierce Biotechnology, Rockford, IL). After 120 min, the reaction was quenched by the addition of 2% formic acid. The acidified digests were further cleared using reverse-phase solid phase extraction (SPE) and washed with 20% MeOH/1% formic acid. The Aβ species were eluted from the SPE plate by the addition of 55% ACN/1% formic acid, dried under vacuum, and stored (− 80 °C) until analysis.

### Plasma Aβ quantification using LC-MS/MS

The dried Aβ peptide digests were reconstituted in 16 μL of 10% ACN/10% formic acid. Three μL of the sample were injected onto a monolithic divinylbenzene column (Thermo Fisher Scientific) and separated using a Waters Acquity M-class UPLC (Waters Corporation, Milford, MA). The Aβ peptides were resolved using a linear gradient of 99.9% water/0.1% formic acid and 99.9% acetonitrile/0.1% formic acid over 6 min. The Aβ peptides were detected using a Thermo Scientific Fusion Lumos Tribrid MS (Thermo Fisher Scientific, Waltham, MA). For the targeted analytes, precursor and product ions were detected using parallel reaction monitoring (PRM). Precursor ions were filtered by quadrupole isolation of 1.6 m/z and detected within the orbitrap at a mass resolution of 30,000. Automatic gain control (AGC) targets for Aβ40 and Aβ42 were set at 1.0e^5^ and 5.0e^5^, respectively. Maximum injection times for all Aβ species was set at 75 msec.

### Aβ isoform quantification

Calibration curves for both Aβ40 and Aβ42 were created by dividing the integrated peak area for each targeted ^14^N-peptide over the integrated peak area for the corresponding ^15^N-peptide, and the measured ^14^N/^15^N ratio for each (*n* = 7) calibrator’s known Aβ40 and Aβ42 peptide concentration were plotted. Linear regression analysis (1/x weighting) was used to determine the correlation coefficient (r^2^ > 0.995), slope and intercept for each calibration curve. Acceptance criteria were established for calibration curve metrics, chromatographic peak retention time, symmetry, intensity, and peptide ion ratios (details provided in Supplementary Information); these were assessed in each sample batch using TraceFinder 4.1 General Quan software (Thermo Fisher Scientific, Waltham, MA). Plasma Aβ40 and Aβ42 concentrations in participant samples and quality control samples were calculated by comparing each sample’s ^14^N/^15^N ratio for Aβ40 and Aβ42 peptides to their respective calibration curves. Ion ratios for the participant samples and quality control samples were within ±20% of the average ion ratios for the seven calibrators. Quality control plasma samples with known Aβ42 and Aβ40 concentrations and Aβ42/40 ratios were analyzed in each batch and were required to pass multi-rule acceptance criteria for their assigned values. For the quality control samples, a 1–3 s rule was implemented where a batch would fail if Aβ40, Aβ42, or Aβ42/40 exceeded the nominal concentration by ±3SD.

### Plasma ApoE qualitative assay internal standard peptides

Stable isotope labeled ApoE internal standard (ISTD) peptides corresponding to the four tryptic ApoE peptide sequences (CLAVYQAGAR, LAVYQAGAR, LGADMEDVCGR, and LGADMEDVR) that distinguish among the six ApoE genotypes were synthesized with uniformly labeled ^13^C,^15^N-arginine at the C-terminus (Vivitide, Gardner, MA).

### Plasma ApoE sample preparation

Plasma samples were prepared using a method modified from van den Broek et al. [32]. Briefly, plasma (5 μL) was diluted into 95 μL 100 mM Tris pH 8.1, 9.6 mM sodium deoxycholate (SDC), 2.3 mM tris(2-carboxyethyl) phosphine (TCEP)). Eight μL of diluted sample were added to 40 μL of 100 mM Tris pH 8.1, 9.6 mM SDC, 2.3 mM TCEP, 10 fmol ISTD peptides/μL. Samples were denatured by incubation at 50 °C with shaking. Proteins were alkylated by the addition of 20 μL of 4.8 mM iodoacetamide and incubated at room temperature, in the dark, with shaking. Proteins were digested by the addition of 24 μL of 0.06 μg/μL mM trypsin (Gold, MS Grade; Promega Corporation, Madison, WI) and incubated at 50 °C with shaking. After digestion, samples were acidified with the addition of 7 μL of 35.7% formic acid/71.4 mM heptafluorobutyric acid (HFBA) to precipitate the SDC. Samples were centrifuged at room temperature to pellet the SDC, and further cleaned using SPE. The samples were dried under vacuum and stored (− 80 °C) until LC-MS/MS analysis.

### ApoE peptide analysis and identification using LC-MS/MS

The dried ApoE peptide digests were reconstituted, 3 μL of the sample was injected onto a CSH C18 column and were separated using a Waters Acquity M-class UPLC. The ApoE peptides were resolved using a linear gradient of 99.9% water/0.1% formic acid and 99.9% acetonitrile/0.1% formic acid over 4 min. The Aβ peptides were detected using a Thermo Scientific Fusion Lumos Tribrid MS (Thermo Fisher Scientific, Waltham, MA). For the targeted analytes, precursor and product ions were detected using parallel reaction monitoring (PRM). Precursor ions were filtered using a quadrupole isolation window of 1.6 m/z. Product ions were detected within the orbitrap at a mass resolution of 30,000 and the AGC target was set to 5.0 × 10^5^.

### ApoE Proteotyping

ApoE proteotyping for each of the six ApoE genotypes (ε2/2, ε2/3, ε2/4, ε3/3, ε3/4, ε4/4) used a combination of the presence or absence of the four targeted ApoE isoform-specific tryptic peptides. The presence or absence of an ApoE isoform-specific peptide was determined by inspecting ratio dot product (rdotp) scores provided by Skyline between the measured transitions for the endogenous and corresponding ISTD peptides [33]. A high rdotp value was used to confirm the ApoE peptide identity in the sample rather than solely relying on limits of detection for the measurands as the threshold for determining presence or absence in a sample. Rdotp values range from 0 to 1, with a score of 0 representing the least amount of endogenous ApoE isoform-specific peptide agreement with the corresponding internal standard peptide and 1 being identical agreement. ApoE peptides with rdotp values greater than or equal to 0.99 were considered present and those with rdotp values less than 0.99 were considered absent. ApoE proteotype was determined using the presence/absence of ApoE isoform-specific peptides as outlined in the Supplementary Information. An in-house R script was utilized to generate the ApoE proteotypes based upon the input peak areas (or their absence) for each isoform-specific peptide.

### Statistical analysis

All data were analyzed using R version 4.0.0 (The R Foundation for Statistical Computing, https://www.r-project.org/). Receiver operating characteristic (ROC), sensitivity, and specificity were calculated using the pROC package for R and optimal cut off values for plasma Aβ42/40 ratio and model parameters were determined by Youden index (maximized sensitivity and specificity of the predictive test) [34]. ROC curve 95% confidence intervals (CI) and comparisons between ROCs were calculated using the DeLong method [35].

Comparisons between groups with only two outcomes were performed using an unpaired two-sided t-test. Comparisons between groups with more than two outcomes were performed using a one-way ANOVA followed by Tukey multiple pairwise-comparisons between the group means. Fisher’s Exact test was used to compare demographic variables (e.g., % women vs. men) between amyloid positive vs. negative groups.

Binary logistic regression models were used to account for cohort heterogeneity across the different banked sample sets, as well as ApoE proteotype, and age as variables for predicting amyloid positivity. Logistic regression models used amyloid positivity as the dependent variable and the cohort, plasma Aβ42/40 ratio, ApoE proteotype, and age as independent variables. ApoE proteotype was included as a dummy variable indicating the number of ApoE4 alleles as either zero, one or two, to allow for non-linear influence of two copies vs. one copy of ApoE4.

## Results

### Cohort demographics

As expected, each cohort included a different number of participants (*n* = 26–121) with differing distributions by sex (23–58% male), age (45–93 years), prevalence of brain amyloid positivity (17–52%), ApoE4 status (19–35% E4 heterozygotes; 4–17% homozygotes), and plasma Aβ42/40 ratios (0.059–0.148; Table 1). Clinical diagnosis was only available for cohort 2, and in this cohort clinical diagnosis aligned with the CDR scores, with around 54% classified as normal, and the remaining receiving a diagnosis of either dementia-AD or MCI-AD. For all but one cohort either MMSE or CDR scores were available (Table 1). Cohorts 1, 2, and 4 were evenly balanced, with around 50% having either CDR or MMSE scores in the normal range, whereas cohorts 3 and 6 had a majority of clinically normal participants.

Brain amyloid status was defined using site-specific methods and cutoff values for determining CSF Aβ42/40 ratio or amyloid PET positive or negative, further breakdown of the demographics for the 6 cohorts by amyloid status can be found in Supplementary Information. These methods varied between and within cohorts and included differences in imaging agent and methods for evaluating images. Using these site-dependent criteria, 161 (39%) of the 414 participants were classified as brain amyloid positive.

Likewise, site-specific procedures were used to collect and process blood, aliquot and store plasma samples, and these varied among the cohorts. Pre-analytical sample handling conditions affect plasma Aβ42 and Aβ40 concentrations [36], but these conditions could not be controlled in this retrospective sample analysis. These heterogeneous conditions allowed us to test the robustness and analytical viability of the sample preparation methods and the LC-MS/MS assay developed, and the practical applicability of the findings among diverse sites, participants, and samples collected under real-world, non-optimal conditions.

After analysis, all plasma Aβ42, Aβ40, Aβ42/40 ratio and ApoE proteotype data were secured, sites shared the corresponding demographic and phenotypic data for statistical analysis with the plasma biomarker data. Initially, the plasma biomarker values were compared between the brain amyloid positive and negative participants as defined by the site-specific method (Table 2). Overall, the amyloid positive group was older (74 vs. 68 yr; *p* < 0.0001), had fewer women (51% vs. 60%; *p* = 0.01), fewer participants with CDR = 0 (32% vs 80%; *p* < 0.0001), fewer participants with normal MMSE of 27–30 (61% vs. 95%; *p* < 0.0001), and more ApoE4 heterozygotes (51% vs. 22%) and homozygotes (17% vs. 7%) than the amyloid negative group. Hispanic/Latino representation (24% vs. 22%; *p* = 0.57) and years of education (16 vs. 16 yr; *p* = 0.41) were not significantly different between the groups. A previous study reported lower CSF biomarker levels among African American ApoE4 carriers than non-Hispanic White ApoE4 carriers [37]. Information about race was available from 4 out of the 6 cohorts (missing from cohort 4 and 5). From this it is clear that the overall racial diversity in the cohorts was low, with an overall preponderance of white research participants.

**Table 2:** Participant characteristics separated by brain amyloid status

	Amyloid Negative (***N*** = 253)	Amyloid Positive (***N*** = 161)
**Age (*P*** **< 0.0001)**		
Mean (SD) [N]	67.7 (8.1) [253]	73.6 (7.4) [161]
**Sex (*P*** **= 0.0139)**		
Female [N]	63.6% [253]	50.9% [161]
**Ethnicity (*P*** **= 0.5734)**		
Hispanic [N]	21.7% [198]	24.5% [110]
**Race**		
White	176	97
Black or African American	8	5
Asian	2	0
American Indian or Alaska Native	1	1
**Education (*P*** **= 0.4080)**		
Mean years (SD) [N]	16.3 (2.4) [170]	16.1 (2.5) [85]
**ApoE4 distribution within subgroup**		
No E4 [N]	71.5% [181]	32.3% [52]
One E4 [N]	21.7% [55]	50.9% [82]
Two E4 [N]	6.7% [17]	16.8% [27]
**CDR (*P*** **< 0.0001)**		
Percent CDR = 0 [N]	79.8% [84]	32% [75]
**MMSE (*P*** **< 0.0001)**		
Percent MMSE = 27–30 [N]	95.4% [152]	60.6% [71]
mean (sd)	29.4 (1.6)	26.2 (4.5)
**Abeta42/40 (*P*** **< 0.0001)**		
mean (sd)	0.101 (0.010)	0.090 (0.010)
min	0.072	0.059
max	0.126	0.148
**Abeta42 (pg/mL) (*P*** **< 0.0001)**		
mean (sd)	44.477 (8.637)	40.421 (9.698)
min	10.984	23.985
max	82.494	103.882
**Abeta40 (pg/mL) (*P*** **= 0.22)**		
mean (sd)	440.435 (81.870)	452.325 (103.933)
min	134.985	270.455
max	893.672	1219.238

*N* Number of total observations - not all demographic data were available from each cohort, so [N] is the number of participants where data was available/provided

### Plasma amyloid isoform concentrations

The sample preparation methods and LC-MS/MS analytical platform developed were sensitive and specific for quantifying plasma Aβ40 and Aβ42 concentrations. The limit of detection (LOD) was 11 pg/mL for Aβ40 and 3 pg/mL for Aβ42. The specificity derives from the ability of tandem mass spectrometry to detect the amino acid sequence for each targeted peptide and fragment ions based on their mass-to-charge ratios. The LC-MS/MS assay has undergone extensive analytical validation to ensure consistent performance over a wide range of Aβ peptide concentrations and all ApoE genotypes (manuscript under review).

**Fig. 1:**
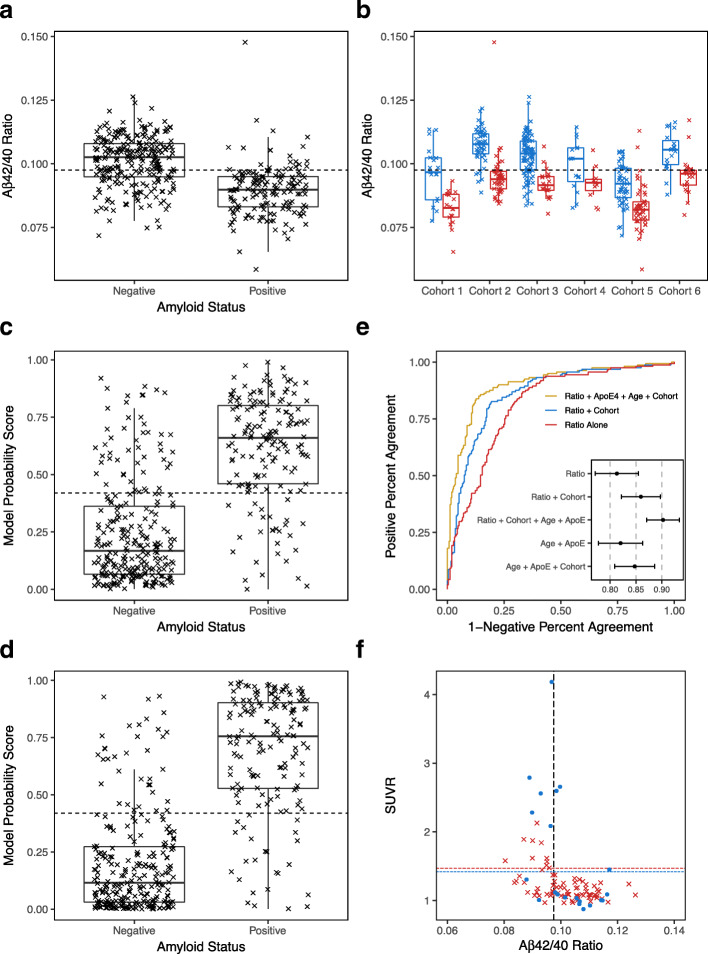
Diagnostic Performance Plots, Metrics, and Prediction Parameters for Plasma Biomarkers Measured Using LC-MS/MS. **a** Plasma Aβ42/40 concentration ratios were lower in amyloid positive than negative participants (*n* = 414). Scatter-Box-Whisker plot of plasma Aβ42/40 for participants classified as brain amyloid negative or positive. Optimal plasma Aβ42/40 cutoff value (0.0975) = dashed horizontal line; Median = dark horizontal lines; 25th to 75th quartiles = Box; 95% Confidence Interval = Whisker. **b** In each cohort, plasma Aβ42/40 ratios were consistently lower in amyloid positive than negative participants. Plasma Aβ42/40 ratios separated by brain amyloid status (Blue = Negative; Red = Positive) for each cohort. Dashed horizontal line is the optimal plasma Aβ42/40 cutoff value (0.0975) based on ratio alone (same as dashed line in **a**). **c** Amyloid probability scores were higher in amyloid positive than negative participants. A logistic regression model using plasma Aβ42/40 and cohort to generate a model probability score that predicted brain amyloid status. Scatter-Box-Whisker plots of individual probability scores (0.0–1.0) separated by amyloid status. Optimal model-derived probability score that differentiated amyloid positive from negative (0.42) = dashed horizontal line. **d** Amyloid probability scores derived from a logistic regression model that used plasma Aβ42/40, number of ApoE4 alleles, age and cohort to predict brain amyloid status. Scatter-Box-Whisker plots of individual amyloid probability scores (0.0–1.0) separated by amyloid status. **e** Receiver Operating Characteristic curves (ROC) plotted using: participants’ plasma Aβ42/40 ratio, ApoE4, age, and cohort (gold plot; AUC-ROC = 0.90 and 95% CI shown in insert); plasma Aβ42/40 and cohort (blue plot; AUC-ROC = 0.86); and only plasma Aβ42/40 (red plot; AUC-ROC = 0.81). For comparison, the insert also shows AUC-ROC and 95% CI for ApoE4 and age (0.82), and ApoE4, age and cohort (0.84). **f** Four-quadrant plot illustrating the relationship between quantitative PiB SUVR values and plasma Aβ42/40 ratios and cutoff value (dashed vertical line = 0.0975) for two cohorts (*n* = 103). Cohort 3 used PiB SUVR cutoff = 1.47 (Red (x) and dashed horizontal line), cohort 6 used 1.42 (Blue filled dots (**•**) and dashed horizontal line). Three false negative plasma Aβ42/40 results in the upper right quadrant. Twenty false positive plasma Aβ42/40 results in the lower left quadrant that may represent participants with elevated risk for converting to amyloid PET positive in the future

The plasma Aβ42 and Aβ40 concentrations were expressed as a concentration ratio (Aβ42/40). As is the case for CSF, this ratio is a better predictor of the presence of brain amyloid than just the plasma Aβ42 concentration, and a lower plasma Aβ42/40 ratio has been associated with amyloidosis [17, 21]. Overall, plasma Aβ42/40 was 11.4% lower in the amyloid positive than in the amyloid negative group (*p* = 2.6 × 10^− 26^) (Fig. 1a). By performing ROC analysis, the plasma Aβ42/40 cutoff value that maximized sensitivity and specificity (Youden index) was found to be 0.0975 (dashed line in Fig. 1a). The area under the ROC curve (AUC-ROC) was 0.81 (95% CI = 0.77–0.85, Fig. 1e, red line) and the overall percent accuracy between the plasma Aβ42/40 ratio and amyloid positivity was 75% at this cutoff value.

Plotting the plasma Aβ42/40 ratios by cohort shows how cohort-specific differences for defining amyloid positivity, and differences in blood collection and storage protocols affected the measured plasma Aβ42/40 ratios (Fig. 1b). Across all cohorts, the plasma Aβ42/40 ratio was consistently lower in the amyloid positive than the amyloid negative participants, but the optimal plasma Aβ42/40 cutoff value appeared to differ slightly among cohorts. A logistic regression model was constructed using plasma Aβ42/40 ratio and cohort as variables to adjust for the cohort differences and their performance for predicting amyloid status independent of any global cohort-specific differences in plasma Aβ42/40 ratios. This logistic regression model provided an overall improvement in AUC-ROC to 0.86 (95% CI = 0.82–0.90, Fig. 1e, blue line) and accuracy to 81%. The logistic regression model generated a probability score (0.0–1.0) for predicting brain amyloid status for each participant; low probability scores predicted a negative amyloid status while high probability scores predicted a positive amyloid status (Fig. 1c). The ROC curve analysis used to calculate model performance generated an optimal (Youden) probability score cutoff of 0.42 (dashed line in Fig. 1c), and the mean probability score was higher among amyloid positive than negative participants (*p* = 1.8 × 10^− 43^). The model that accounted for cohort differences in plasma sample collection and processing procedures, along with plasma Aβ42/40 concentration ratios, provided an improvement in overall predictive value for the presence or absence of brain amyloidosis. The AUC-ROC analysis for the model that used plasma Aβ42/40 alone (0.81) was significantly lower (*p* = 0.00031) than the AUC-ROC analysis that used plasma Aβ42/40 ratio and cohort (0.86).

### Inclusion of ApoE and age improves diagnostic performance

ApoE genotype and participant age are established risk factors for Alzheimer’s disease and brain amyloidosis [1]. Along with plasma Aβ40 and Aβ42 quantitation, we developed sample preparation procedures and LC-MS/MS detection methods that identified plasma ApoE isoform-specific peptides expressed by ApoE genes. We refer to this as plasma ApoE proteotyping or phenotyping. The method requires 5 μL plasma and no genetic material. ApoE proteotyping successfully confirmed all participants’ ApoE genotypes as provided in the demographic data from the various cohorts (Table 1).

We examined whether adding participant age and ApoE proteotype offered further improvements to the performance of the logistic regression model’s probability score for predicting brain amyloid status. Age was added to the logistic regression model as a continuous variable, and the number of ApoE4 alleles was added as a non-linear variable. Adding these factors to the logistic regression model improved the separation in probability scores between brain amyloid positive and negative (*p* = 3.2 × 10^− 55^; Fig. 1d) without dramatically altering the optimal cutoff value (0.44), significantly increased (*p* = 0.0010) the AUC-ROC to 0.90 (95% CI = 0.87–0.93: Fig. 1e, gold line) and the overall accuracy to 86%. There was no significant difference in performance of this model between the 6 different cohorts (*p* = 0.47 for Fisher test on accuracy data). For comparison, the AUC-ROC for just age and ApoE4 copy number was only 0.82, and AUC-ROC for age, cohort, and ApoE4 copy number (excluding plasma Aβ42/40 ratio) was 0.84 (Insert in Fig. 1e).

### Plasma Aβ42/40 concentration compared to SUVR

Of the 414 participants, 251 had quantitative amyloid PET SUVR measures. The SUVR data were collected using 3 different tracers: PiB (Pittsburgh Compound B), Amyvid (Florbetapir), or NeuraCeq (Florbetaben) at multiple clinical sites using different scanners, modalities, and non-standardized acquisition parameters (Table 1). While the SUVR values cannot be directly compared among the tracers and sites, when the SUVR values were plotted against their corresponding plasma Aβ42/40 ratios, there was a grouping of SUVR values below the SUVR cutoffs for participants who were amyloid negative by the blood test, while for participants who were positive by the blood test, the SUVR values were either above or below the SUVR cutoff value. This is illustrated in Fig. 1f for PiB (*n* = 103), but similar patterns were observed for the other PET tracers. Quantitative PiB SUVR values were available from cohorts 3 and 6. But, each cohort used slightly different SUVR cutoff values to define amyloid positive and amyloid negative participants (1.47 for cohort 3 and 1.42 for cohort 6), so the data points from the two cohorts are shown as red and blue with color matching horizontal lines denoting the cutoff value for each cohort (Fig. 1f). This plot shows that there are three participants with SUVR values above background uptake who had plasma Aβ42/40 ratios above the cutoff value (false negative by the plasma Aβ42/40 ratio measures). However, 20 participants with SUVR values in the normal range were predicted to be amyloid positive by the plasma Aβ42/40 ratio measures (false positives). This is consistent with what has been observed with CSF Aβ42/40 assays and could suggest that the Aβ42/40 ratio can detect biochemical changes prior to brain amyloid accumulating to the level required for detection by PET imaging.

## Discussion

The findings indicate that the plasma sample preparation and LC-MS/MS analytical methods developed for our blood biomarker assay are robust, analytically viable, practically applicable, and resilient to the potential variability introduced when plasma samples are collected using non-standardized procedures from participants under real-world conditions. The findings provide proof-of-principle; this LC-MS/MS method has strong diagnostic performance and accurately (AUC-ROC 0.81–0.90) distinguished brain amyloid positive from amyloid negative individuals, even when various imaging and CSF fluid biomarker platforms were used as reference standards to determine brain amyloid status.

The diagnostic performance metrics are excellent, considering: i) several different sites provided plasma samples and phenotypic data; ii) the diverse and heterogeneous participant demographics; iii) the difference in methodologies used to identify brain amyloid status; iv) the different methods, definitions, and cutoff values sites used to distinguish brain amyloid positive from negative; and v) the non-standardized site-specific procedures used to collect blood, isolate, aliquot, freeze and store the plasma samples prior to Aβ- and ApoE-isoform specific peptide analysis. Despite these uncontrolled factors, the sample preparation procedure and LC-MS/MS method performed remarkably well. This warrants further investigation to qualify, verify, and validate the analytical and clinical performance parameters in larger cohorts using controlled, consistent protocols and procedures. Such analytical and clinical validation studies are in progress by our group, and planned for regulatory review and clearance (e.g., US FDA, College of American Pathologists).

The findings also indicate that plasma Aβ42/40 concentration ratio determined using the LC-MS/MS assay developed can accurately identify brain amyloid status, and that including additional risk factors for amyloid pathology in the model (age, ApoE4 copy number) improved the AUC-ROC and model accuracy. This is consistent with what has been observed by other groups and is based on established relationships among advanced age, number of ApoE4 alleles and amyloid pathology [17, 23, 38]. It is important to note that the plasma Aβ42/40 ratio combined with ApoE and age improved the accuracy for identifying amyloid positivity as compared to just ApoE and age alone. The AUC-ROC for plasma Aβ42/40 ratio and cohort was 0.86 while the AUC-ROC for ApoE, age, and cohort was 0.84, but when plasma Aβ42/40 ratio, ApoE, age, and cohort were combined into a single model, the AUC-ROC improved to 0.90. This suggests that the risk/susceptibility information provided by the ApoE genotype and age is additive to the biochemical information provided by the plasma Aβ42/40 ratio. At an AUC-ROC of 0.90, the accuracy of the plasma test approaches the upper limit of accuracy for the diverse methods used to determine amyloid status in these cohorts. It is also notable that this LC-MS/MS analytical platform adds efficiency and value to the prediction algorithm by identifying the plasma ApoE proteotype in the same plasma sample. This eliminates the need for a separate blood collection for traditional ApoE genotyping procedures.

Similar to other studies that tested concordance between CSF or plasma Aβ42/40 ratio against amyloid PET status, the current plasma biomarker analyses yielded a greater number of false positive than false negative findings (Fig. 1f), i.e., there were more individuals with a positive plasma biomarker result who had a negative amyloid PET scan than the reverse situation [17, 20, 23]. This is consistent with preliminary findings from a longitudinal study where amyloid PET negative individuals with a low plasma Aβ42/40 ratio had a 15-fold greater risk of converting to amyloid PET positive within 4 years when compared to individuals with a high plasma Aβ42/40 ratio [17]. As suggested, a low plasma Aβ42/40 ratio may identify individuals who will convert from amyloid PET negative to positive in the near future [17], but additional longitudinal evidence is required.

These observations suggest that the plasma Aβ42/40 ratio declines before brain amyloid accumulates to a level that can be detected by currently available PET tracers, and that fluid biomarkers and amyloid PET SUVR reflect different aspects/stages of amyloid pathology. Fluid biomarkers might provide an earlier indication of changes in Aβ42 (and other soluble neuroproteins) production and clearance rates, while amyloid PET SUVR reflects the consequence of these kinetic changes in the form of accumulation of neuritic amyloid plaques that take many years to evolve. Large longitudinal studies are needed to better elucidate the relationships among changes in CSF and plasma Aβ42/20 ratio, and amyloid PET SUVR values.

The use of banked samples from multiple diverse cohorts provides proof-of-principle for the analytical robustness of the LC-MS/MS assay. However, the lack of consistent enrollment criteria, sample collection methods, and amyloid status definitions are limitations as these factors may have introduced a sample selection bias, and they do not allow cohort-independent cutoff values to be established for plasma Aβ42/40 ratio or amyloid probability score. The Plasma test for Amyloid Risk Screening (PARIS) study, a C_2_N sponsored prospective and controlled study, enrolled patients with amyloid PET imaging obtained as part of the IDEAS (Imaging Dementia Evidence for Amyloid Scanning) study [39], was recently completed and tested concordance between plasma Aβ42/40, ApoE, age and amyloid PET status (central read) in patients who met the IDEAS inclusion criteria (NCT02420756). The first phase of the PARIS study established cutoff values (manuscript in preparation) for the now available PrecivityAD™ CLIA test (C_2_N Diagnostics, St. Louis, MO).

The lack of consistent enrollment criteria among the six cohorts may also have contributed to the unusually good performance of the model that included only ApoE4 copy number and age to predict amyloid status. This model had an AUC-ROC of 0.84 when cohort was included, whereas such models normally have AUC-ROCs in the 0.75–0.80 range [23, 24]. However, when plasma Aβ42/40 ratio was included in this model, the model performance (AUC-ROC) increased (Fig. 1e), supporting the notion that the plasma Aβ42/40 ratio captures unique biological information that is additive to the ApoE and age information.

Analytical validation metrics for this LC-MS/MS assay that conformed to the standards of Clinical Laboratory Improvement Amendments (CLIA) were presented at the virtual 2020 CTAD meeting [40]. This, combined with the clinical performance metrics presented (Fig. 1) prompted C_2_N to complete the first phase of the PARIS study, clinically validate (CLIA) the PrecivityAD™ test, and release it for use in the clinic to aid clinicians evaluating individuals experiencing early cognitive impairment. Full analytical and clinical validation, according to FDA’s in vitro diagnostic (IVD) regulations, are underway.

## Conclusions

The LC-MS/MS analytical platform and algorithm presented here constitute a test that can accurately identify brain amyloid status based on a single blood sample. Despite differences in how each cohort site collected and stored plasma samples, and defined presence or absence of brain amyloid, the findings indicate that this blood test and logistic regression model that incorporates plasma Aβ42/40, ApoE4 status, and patient age had excellent performance; AUC-ROC = 0.90 when compared to CSF or amyloid PET biomarkers. Brain PET imaging or CSF biomarker analysis currently represent the primary approaches used to identify AD pathological changes in living individuals. However, amyloid PET is resource-intensive, costly, and exposes individuals to unnecessary radiation. The CSF biomarkers require invasive sampling that deters many individuals from undergoing such testing. There is an urgent need for non-invasive and easily available diagnostic tools that identify AD pathology. This blood-based test may be a useful aid in the diagnosis of AD, therefore allowing for improved medical decision making and management, streamlined AD clinical trial enrollment, and better identification of who may benefit from an AD specific therapy.

## Supplementary Information

**Additional file 1: Table S1.** Acceptance criteria of Aβ 40 and Aβ 42 assays. **Table S2.** ApoE proteotype determination based on present/absent of call of isoform-specific peptides. **Table S3.** Participant characteristics separated by brain amyloid status, for each cohort.

## Data Availability

The datasets used and/or analyzed during the current study are available from the corresponding author on reasonable request.

## Abbreviations

AD: Alzheimer’s disease
ADRC: Alzheimer’s Disease Research Center
Aβ: Amyloid-beta
CSF: Cerebrospinal fluid
MS: Mass Spectrometry
LC: Liquid chromatography
ApoE: Apolipoprotein E
CDR: Clinical Dementia Rating
MMSE: Mini-Mental State Examination
SUVR: Standard uptake value ratio
PET: Positron emission tomography
PiB: Pittsburgh compound B
AAA: Amino acid analysis
ISTD: Internal standard
PRM: Parallel reaction monitoring
LOD: Limit of Detection
ROC: Receiver operating characteristic
AUC-ROC: Area under the ROC curve
CI: Confidence interval
FDA: US Food and Drug Administration
IVD: In vitro diagnostic
CLIA: Clinical Laboratory Improvement Amendments
SD: Standard deviation
