# Supplementary material for: A blood-based diagnostic test incorporating plasma Aβ42/40 ratio, ApoE proteotype, and age accurately identifies brain amyloid status: findings from a multi cohort validity analysis
Source: Mol Neurodegener. 2021 May 1;16:30. doi: 10.1186/s13024-021-00451-6 (PMC8088704; doi:10.1186/s13024-021-00451-6)
Supplement: Supplementary file 1 — Additional file 1: Table S1. Acceptance criteria of Aβ 40 and Aβ 42 assays. Table S2. ApoE proteotype determination based on present/absent of call of isoform-specific peptides. Table S3. Participant characteristics separated by brain amyloid status, for each cohort. [file 13024_2021_451_MOESM1_ESM.docx]

Additional file 1 -- Supplementary Information:

Table S1. Acceptance criteria of Aβ 40 and Aβ 42 assays.

| Parameter | Acceptance Limit |
| --- | --- |
| ISTD Peak Area recovery (to mean Calibrator ISTD Peak Area) | ± 50% |
| Relative Retention Time (min) to ISTD | ± 2.5% |
| Chromatographic Peak Symmetry at Half Height | ≥ 65% |
| Peak Resolution (min) | ≥ 0.200 |
| Peak Width at Half Height (sec) | 1.25 - 4.50 |
| Ion Ratios (as compared to mean Calibrator ion ratios) | ± 20% |
| Calibrator accuracy from nominal concentration | ± 10%* |

*Except the lowest calibrator which is set at ± 15%

Table S2. ApoE proteotype determination based on present/absent of call of isoform-specific peptides.

| **CLAVYQAGAR** | **LAVYQAGAR** | **LGADMEDVCGR** | **LGADMEDVR** | **ApoE Proteotype** |
| --- | --- | --- | --- | --- |
| Present | Absent | Present | Absent | E2/E2 |
| Present | Present | Present | Absent | E2/E3 |
| Present | Present | Present | Present | E2/E4 |
| Absent | Present | Present | Absent | E3/E3 |
| Absent | Present | Present | Present | E3/E4 |
| Absent | Present | Absent | Present | E4/E4 |

Table S3 Participant characteristics separated by brain amyloid status, for each cohort.

|  | Cohort 1, Amyloid Negative | Cohort 1, Amyloid Positive | Cohort 2, Amyloid Negative | Cohort 2, Amyloid Positive | Cohort 3, Amyloid Negative | Cohort 3, Amyloid Positive | Cohort 4, Amyloid Negative | Cohort 4, Amyloid Positive | Cohort 5, Amyloid Negative | Cohort 5, Amyloid Positive | Cohort 6, Amyloid Negative | Cohort 6, Amyloid Positive |
| --- | --- | --- | --- | --- | --- | --- | --- | --- | --- | --- | --- | --- |
| **N** | 19 | 18 | 51 | 43 | 100 | 21 | 15 | 11 | 49 | 47 | 19 | 21 |
| **Age, mean (sd)** | 69.4 (7.2) | 77.0 (6.4) | 69.6 (7.6) | 72.8 (8.2) | 65.2 (8.1) | 70.4 (4.5) | 72.6 (4.7) | 78.5 (7.9) | 70.2 (7.4) | 72.6 (7.6) | 64.0 (8.3) | 74.9 (6.3) |
| **Sex (% male)** | 47.4% | 61.1% | 58.8% | 58.1% | 23% | 23.8% | 46.7% | 72.7% | 38.8% | 42.6% | 21.1% | 47.6% |
| **Biomarker Used to Determine**  **Brain Amyloid Status (% participants)** | | | | | | | | | | | | |
| PIB | - | - | - | - | 72% | 47.6% | 73.3% | 27.3% | - | - | 68.4% | 38.1% |
| Amyvid | - | - | - | - | 28% | 52.4% | - | - | 20.4% | 23.4% | 31.6% | 61.9% |
| Neuraceq | - | - | - | - | - | - | - | - | 79.6% | 76.6% | - | - |
| CSF ELISA | - | - | 100% | 100% | - | - | - | - | - | - | - | - |
| CSF IPMS | 100% | 100% | - | - | - | - | 26.7% | 72.7% | - | - | - | - |
| **ApoE Proteotype** |  |  |  |  |  |  |  |  |  |  |  |  |
| E2/E3 | 10.5% | - | 11.8% | 4.7% | 6% | - | 40% | - | 10.2% | 4.3% | 10.5% | 4.8% |
| E2/E4 | - | 5.6% | 5.9% | 7% | - | - | - | - | 2% | 2.1% | 5.3% | 4.8% |
| E3/E3 | 84.2% | 16.7% | 64.7% | 25.6% | 53% | 23.8% | 60% | 27.3% | 67.3% | 36.2% | 52.6% | 38.1% |
| E3/E4 | 5.3% | 55.6% | 15.7% | 44.2% | 26% | 47.6% | - | 45.5% | 18.4% | 51.1% | 31.6% | 38.1% |
| E4/E4 | - | 22.2% | 2% | 18.6% | 15% | 28.6% | - | 27.3% | 2% | 6.4% | - | 14.3% |
| **C_2_N Plasma Aβ42/40, mean (sd)** | 0.096 (0.011) | 0.082 (0.007) | 0.107 (0.007) | 0.095 (0.010) | 0.103 (0.008) | 0.092 (0.006) | 0.099 (0.010) | 0.092 (0.007) | 0.092 (0.008) | 0.083 (0.009) | 0.105 (0.008) | 0.095 (0.008) |
